# A Drug Screening Reveals Minocycline Hydrochloride as a Therapeutic Option to Prevent Breast Cancer Cells Extravasation across the Blood–Brain Barrier

**DOI:** 10.3390/biomedicines10081988

**Published:** 2022-08-16

**Authors:** Joana Godinho-Pereira, Margarida Dionísio Lopes, Ana Rita Garcia, Hugo M. Botelho, Rui Malhó, Inês Figueira, Maria Alexandra Brito

**Affiliations:** 1 iMed—Research Institute for Medicines, Faculty of Pharmacy, Universidade de Lisboa, Av. Prof. Gama Pinto, 1649-003 Lisbon, Portugal; 2 Department of Pharmaceutical Sciences and Medicines, Faculty of Pharmacy, Universidade de Lisboa, Av. Prof. Gama Pinto, 1649-003 Lisbon, Portugal; 3 BioISI—Biosystems and Integrative Sciences Institute, Faculty of Sciences, Universidade de Lisboa, Campo Grande, 1746-016 Lisbon, Portugal; 4 Farm-ID—Faculty of Pharmacy Association for Research and Development, Av. Prof. Gama Pinto, 1649-003 Lisbon, Portugal

**Keywords:** β-catenin, blood–brain barrier, breast cancer brain metastases, buparlisib, extravasation, microRNAs, minocycline hydrochloride, preventive approach, phosphoinositide 3-kinase inhibitors, tetracyclines

## Abstract

Among breast cancer (BC) patients, 15–25% develop BC brain metastases (BCBM), a severe condition due to the limited therapeutic options, which points to the need for preventive strategies. We aimed to find a drug able to boost blood–brain barrier (BBB) properties and prevent BC cells (BCCs) extravasation, among PI3K, HSP90, and EGFR inhibitors and approved drugs. We used BCCs (4T1) and BBB endothelial cells (b.End5) to identify molecules with toxicity to 4T1 cells and safe for b.End5 cells. Moreover, we used those cells in mixed cultures to perform a high-throughput microscopy screening of drugs’ ability to ameliorate BBB properties and prevent BCCs adhesion and migration across the endothelium, as well as to analyse miRNAs expression and release profiles. KW-2478, buparlisib, and minocycline hydrochloride (MH) promoted maximal expression of the junctional protein β-catenin and induced 4T1 cells nucleus changes. Buparlisib and MH further decreased 4T1 adhesion. MH was the most promising in preventing 4T1 migration and BBB disruption, tumour and endothelial cytoskeleton-associated proteins modifications, and miRNA deregulation. Our data revealed MH’s ability to improve BBB properties, while compromising BCCs viability and interaction with BBB endothelial cells, besides restoring miRNAs’ homeostasis, paving the way for MH repurposing for BCBM prevention.

## 1. Introduction

About 2.3 million new cases of breast cancer (BC) are diagnosed every year, being the most common cancer amongst women [1]. It is predicted that by 2050, female BC will reach 3.2 million new cases per year [2]. BC is the second most frequent cause of brain metastases, and it is estimated that about 15–25% of BC patients will develop this poor prognosis disorder with 1-year survival rates of 20% [3,4]. This scenario is aggravated within the triple negative BC (TNBC) subtype, which often gives rise to grade III tumours with an aggressive phenotype, highly associated with BC brain metastases (BCBM) development [2,5]. When BCBM are diagnosed, the clinical options available are whole brain radiotherapy, stereotactic radiosurgery, surgical resection, chemotherapy, and targeted therapies [6,7,8]. However, therapeutic options face the obstacle of the blood–brain barrier (BBB) [6], which prevents the entrance into the brain of around 98% of drugs [9].

The BBB is an interconnected, continuous monolayer of polarised endothelial cells with elaborate junctional complexes, formed by proteins such as claudin-5 and zonula occludens (ZO)-1 of the tight junctions (TJs) and β-catenin from the adherens junctions (AJs) [9]. Despite being highly impermeable, the BBB enables the entrance of cancer cells into the central nervous system (CNS) [9,10,11], causing metastases establishment. As far as brain metastases are concerned, it is known that metastatic cells can breach the BBB, causing disruption of the junctional complexes and compromising the integrity of the barrier [12,13]. 

Several pathways are deregulated in cancer cells, where kinases play important roles. One of the most altered kinases in human cancer is the phosphoinositide 3-kinase (PI3K), whose signalling pathway has been subject of intense investigation in cancer research [14,15]. Due to the increasing evidence of the role of PI3K in oncogenesis and metastases development, many inhibitors have been developed [16]. However, the use of PI3K inhibitors as a metastases prevention approach is still unexplored, and many inhibitors, including buparlisib (BKM120) [17], may be promising. This is highlighted by buparlisib’s anti-tumour effect [18], particularly its role in reducing BC metastases in organs such as the brain [19]. Fingolimod (or FTY720) is an immunomodulator, specifically a sphingosine-1-phosphate receptor modulator, approved as therapy for patients with relapsing forms of multiple sclerosis [20]. It inhibits endothelial activation via inhibition of nuclear factor (NF)-κB and PI3K, which consequently leads to decreased expression of cell adhesion molecules and to the prevention of leukocyte recruitment during CNS inflammation [20]. It is known that, similarly to leukocytes, tumour cell extravasation across the BBB is dependent on interactions between adhesion molecules expressed in both endothelial and tumour cells [21]. Moreover, FTY720-P, the biologically active form, increased the expression of claudin-5 and enhanced the transendothelial electrical resistance (TEER) in brain microvascular endothelial cells (BMECs) exposed to sera of patients with multiple sclerosis, indicating its ability to prevent the BBB disruption in this pathological condition [22]. These observations raise the hypothesis that FTY720 may improve BBB properties and block BC cells (BCCs) extravasation across BMECs, which might represent a novel approach to prevent BCBM formation. KW-2478, a heat-shock protein 90 (HSP90) inhibitor, has been proven to have anti-tumour activity in models of multiple myeloma [23,24]. In fact, HSP90 is responsible for the stabilisation of several oncogenic kinases such as PI3K or tyrosine kinases (Src, or MAPK) [25,26] and its inhibition could be of importance to hinder the metastatic capacity of tumour cells. Nevertheless, this drug’s potential is largely understudied, both in other cancer types and in prevention of metastasis formation. Canertinib (also known as CI-1033) is an irreversible inhibitor of the four members of the epidermal growth factor receptor (EGFR) tyrosine kinases, shown to inhibit tumour cell growth, and to induce cell apoptosis in several cancer models, such as melanoma and BC [27,28]. However, canertinib’s role in the BBB and its potential to hinder tumour cells extravasation are still undetermined, which could be of interest to explore. On the other hand, tetracyclines are broad-spectrum antibiotics commonly used in clinical practice [29] with acknowledged brain protective effects, particularly at the BBB level, as described for minocycline during cerebral ischemia [30,31]. Moreover, minocycline effectively crosses the BBB and has neuroprotective potential in CNS injury, stroke, and neurodegenerative diseases [32,33,34]. Importantly, minocycline has also been linked to tumour growth inhibition in human prostate and ovarian cancer cell lines and in mice xenograft models [35,36], suggesting an additional effect of this drug. Therefore, tetracyclines emerge as candidates for modulation of BBB properties and prevention of BCBM, deserving to be further addressed. 

Recent work by our team identified several microRNAs (miRNAs or miRs), such as miR-194-5p and miR-205-5p, which are aberrantly expressed in plasma throughout the development of BCBM [37]. These observations point to those miRNAs as circulating biomarkers of BCBM formation and of efficacy monitoring of therapeutic strategies. Interestingly, our subsequent in vitro studies revealed that miR-194-5p and miR-205-5p are expressed and released by BCCs and BMECs and during their interaction [38], mirroring the phenotype observed in vivo [37]. Thus, we hypothesised that miRNAs expression profiles in cell cultures may reflect the alterations occurring in injurious conditions and in response to drug treatment.

In this work, we aimed to disclose a pharmacological modulator to prevent BCCs extravasation by improving BBB properties and counteracting malignant cell features, ultimately hindering BCBM formation. To this end, we performed a high-throughput screening (HTS) of potential drug candidates, including several PI3K, HSP90, and EGFR inhibitors discovered based on a computer-assisted drug discovery campaign [39], and clinically approved drugs, such as fingolimod and some tetracyclines. Using an improved in vitro model of the BBB formed by confluent monolayers of mouse BMECs (b.End5) in conditions mimicking physiologic shear stress [40], we performed automated fluorescence microscopy to monitor BBB alterations upon exposure to TNBC cells (4T1), and their prevention by the selected drugs. Two PI3K inhibitors and one tetracycline revealed the capacity to avoid the injury caused by tumour cells on membrane β-catenin expression at the BBB level, simultaneously increasing the number of 4T1 cells with aberrant nuclei. Minocycline hydrochloride (MH) emerged as the most promising molecule studied, being able to decrease tumour cells’ migration and adhesion to the BBB endothelium, as well as to prevent the formation of endothelial membrane gaps and monolayer holes. Moreover, this drug prevented both BMECs and tumour cells’ cytoskeleton-associated protein alterations and counteracted BCBM-related alterations in specific miRNAs release and cellular expression, opening new doors for this tetracycline’s repurposing for BCBM prevention.

## 2. Materials and Methods

### 2.1. Cell Culture Conditions

#### 2.1.1. Culture of Endothelioma Cell Line

The mouse BALB/c brain endothelioma cell line b.End5 (ECACC, Salisbury, UK) was used as a simplified BBB in vitro model [41]. b.End5 cells were grown in Dulbecco’s modified Eagle medium (DMEM high glucose, #41966052, Gibco, Life Technologies, New York, NY, USA) supplemented with 10% (*v*/*v*) foetal bovine serum (FBS, Sigma Aldrich, St. Louis, MO, USA), 1% (*v*/*v*) non-essential amino acids (Biochrom AG, Berlin, Germany), 2 mM _L_-glutamine (Biochrom AG, Berlin, Germany), 1 mM sodium pyruvate (Biochrom AG, Berlin, Germany), and 1% (*v*/*v*) antibiotic–antimycotic solution (Sigma Aldrich, St. Louis, MO, USA). Cells were maintained at 37 °C in humid atmosphere enriched with 5% CO_2_. For the viability assay, b.End5 cells were seeded using a volume of 200 µL at a density of 2.5 × 10^4^ cells/mL in rat tail collagen-I (100 µg/mL)-coated 96-well plates and incubated for 48 h at 37 °C and 5% CO_2_.

#### 2.1.2. Culture of TNBC Cell Line

The murine mammary carcinoma triple negative 4T1 cell line (ATCC, Middlesex, UK) was used. The 4T1 cells were cultured in RPMI 1640 (Sigma Aldrich, St. Louis, MO, USA) supplemented with 2 mM _L_-glutamine and 5% (*v*/*v*) FBS. Cells were maintained at 37 °C in humid atmosphere enriched with 5% CO_2_. For experiments, 4T1 cells were seeded using a volume of 200 µL at a density of 2 × 10^4^ or 1.5 × 10^5^ cells/mL (viability and wound-healing assay, respectively) in uncoated 96-well plates and incubated for 48 h.

#### 2.1.3. Cell Model of BCBM Formation

As an in vitro model that mimics the interaction between BCCs and BBB endothelial cells preceding BCBM development, mixed cultures of b.End5 and 4T1 cells were used as implemented in our lab [40]. Briefly, b.End5 cells were seeded at a volume of 200 µL at a density of 2.5 × 10^4^ cells/mL in 96-well polystyrene F-bottom cell culture plates (for HTS) or 500 µL at a density of 5 × 10^4^ cells/mL on coverslips in 24-well plates (for immunocytochemistry, ICC, and in situ hybridisation, ISH). Plates were previously coated with rat tail collagen-I (100 µg/mL) and incubated for 48 h. After this time, physiological shear stress (SS) was applied and maintained until the endpoint of each experiment [40]. After 24 h of SS, 4T1 cells previously labelled with CellTracker™ Green CMFDA Dye (5 µM; Thermo Fisher Scientific, Waltham, MA, USA) and resuspended in DMEM were added at a volume of 200 µL at a density of 5 × 10^4^ cells/mL (HTS) or 500 µL at a density of 1 × 10^5^ cells/mL (ICC and ISH) on top of b.End5 confluent monolayers. Mixed cultures were analysed after 24 h. The controls of these cultures were performed in parallel, using single cultures (b.End5 and 4T1 cells alone) and the same experimental conditions. The supernatants of these cultures were collected for miRNAs’ release evaluation by real-time quantitative PCR (RT-qPCR).

### 2.2. Drug Preparation

Information about the molecules used is provided in Supplementary Materials Table S1. PI3K inhibitors (Molecules 1–6), CI-1033, and KW-2478 were acquired from MolPort (MolPort SIA, Riga, Latvia) and BKM120 was kindly provided by MedChemExpress (MedChemExpress, Monmouth Junction, NJ, USA). The tetracyclines MH, minocycline base (MB), doxycycline hyclate (DH), and doxycycline monohydrate (DM) were kindly provided by Dr. Carla Vozone from Hovione (Lisbon, Portugal). FTY720 was purchased from Sigma Aldrich (St. Louis, MO, USA) and FTY720-P from ClearSynth (ClearSynth Labs, Villeurbanne, France). Each drug stock solution was prepared in dimethyl sulfoxide (DMSO, Sigma Aldrich, St. Louis, MO, USA) or in sterile water (for MH and DH), aliquoted and stored at −20 °C, except for buparlisib (BKM120), which was stored at −80 °C. 

### 2.3. Cell Viability Assay

Cell viability was determined by the thiazolyl blue tetrazolium (MTT) assay to infer drug toxicity to 4T1 cells and safety to b.End5 cells. Forty-eight hours after seeding, each cell culture was incubated with the drugs diluted in DMEM at concentrations ranging from 0.1 to 100 µM for the majority of the molecules. According to features of known molecules, lower concentrations were included, namely 0.01 µM for BKM120 and even 0.001 µM for FTY720 and FTY720-P. In parallel, untreated cells (incubated with DMEM only) were used as control. After 24 h, DMEM containing 0.5 mg/mL of MTT (Alfa Aesar, Haverhill, MA, USA) was added to each well. The b.End5 cells were incubated for 3 h, whereas 4T1 cells were incubated for 1.5 h at 37 °C, after which the supernatants were discarded, and the formazan crystals solubilised with a solution of 0.04 N HCl in isopropanol (Honeywell, Charlotte, NC, EUA). Absorbance values were measured using a microplate reader (Zenyth 3100, Anthos Labtec Instruments, Salzburg, Austria) at 595 nm and results presented as percentage relative to untreated cells.

### 2.4. Immunocytochemistry and High-Throughput Screening

BBB integrity indicators, as well as phenotypic alterations in endothelial and tumour cells in single and mixed cultures upon drug treatment, were evaluated by immunofluorescence analysis [40] of the AJs and TJs proteins, β-catenin, and ZO-1, respectively, the phosphorylated form of the cytoskeleton-associated protein myosin light chain (p-MLC), and of the signalling molecule Ras homolog family member A (RhoA). 

The drugs to be tested at the selected concentrations, diluted in DMEM, were applied to both single and mixed cultures (described in Section 2.1) at the time of 4T1 cells’ addition. In parallel, untreated b.End5, 4T1, and mixed cultures, incubated with DMEM only, were used as control. After 24 h, cells were fixed with 4% (*w*/*v*) paraformaldehyde (PFA, Sigma-Aldrich, St. Louis, MO, USA) in phosphate-buffered saline (PBS), permeabilised with 0.3% Triton X-100 (VWR International, Radnor, PA, USA), and blocked with 3% bovine serum albumin (BSA, Sigma Aldrich, St. Louis, MO, USA). Cells were then processed for HTS or conventional immunocytochemistry, as described below, using the antibodies indicated in Table 1, diluted in the blocking solution. Nuclei were counterstained with Hoechst 33342 dye (Thermo Fisher Scientific, Waltham, MA, USA; 20 µM). 

For HTS, cells were incubated with anti-β-catenin antibody for 2 h at room temperature, followed by the incubation of the corresponding secondary antibody for 60 min, at room temperature, and maintained in PBS at 4 °C until image acquisition. Based on this HTS, the most promising drug was selected and its effect on TJs protein and cytoskeleton-associated proteins was evaluated by ICC. To this end, cells were incubated overnight at 4 °C with the primary antibodies and thereafter with the corresponding secondary antibodies for 60 min at room temperature. Methanol-dehydrated cells were then mounted on microscopy slides with DPX (Merck Millipore, Burlington, MA, EUA), properly dried, and stored at 4 °C until image acquisition. Negative control assays were performed without primary antibody.

### 2.5. Wound-Healing Assay

The 4T1 cells migration was evaluated by a wound-healing assay. After 4T1 cultures reached confluence, a longitudinally straight stripe with a constant diameter was made using a 10 µL sterile pipette tip, followed by washing three times with Hank’s balanced salt solution (HBSS, Gibco, Life Technologies, New York, NY, USA). The cells were then incubated in the absence (untreated) or in the presence of each drug, in DMEM, at the selected concentration. Then, 24 or 48 h incubations were performed in the absence of FBS to avoid proliferation, and results are presented as percentage relative to untreated cells.

### 2.6. Real-Time Quantitative PCR

Total RNA from cell culture media was isolated using the miRNeasy Serum/Plasma Advanced Kit (Qiagen, Dusseldorf, Germany), according to the manufacturer’s instructions. RNA was then transcribed into cDNA, using the miRCURY LNA RT Kit (Qiagen, Dusseldorf, Germany), according to the manufacturer’s instructions, though the RNA volume used was increased four times, as previously optimised [37,38]. Prior to the reverse transcription reaction, the synthetic RNA UniSp6 RNA spike-in (Qiagen, Dusseldorf, Germany) was added to the mixture. The reaction was performed on a Biometra T-Combi thermocycler (Analytic Jena, Jena, Germany), using the following conditions: 42 °C for 60 min; 95 °C for 5 min to heat-inactivate the reverse transcriptase, and cooling down and storage at 4 °C.

RT-qPCR was performed using a QuantStudio™ 7 Flex Real-Time PCR System (Applied Biosystems, Waltham, MA, USA) and miRCURY LNA SYBR Green PCR Kit (Qiagen, Dusseldorf, Germany) according to the manufacturer’s instructions using cDNA diluted at 1:6. The following conditions were used: 50 cycles of 95 °C for 15 s, 56 °C for 30 s, 72 °C for 30 s, and a ramp rate of 1.6 °C/s, followed by a melting curve analysis. Predesigned locked nucleic acid (LNA) primer pairs were purchased from Qiagen for each of the selected miRNAs (mmu-miR-194-5p and mmu-miR-205-5p) and miR-16-5p was used as an endogenous control to normalise the expression levels. RT-qPCR was performed in 384-well plates, with each sample analysed in triplicate, and a no-template control was included for each amplification. Determination of the threshold cycle was performed using the QuantStudio™ Real-Time PCR software (Applied Biosystems, Waltham, MA, USA), and the quantifications were performed using the ∆∆Ct method. The results are presented as fold change.

### 2.7. In Situ Hybridisation

ISH was performed as previously described [38] to evaluate the expression of miR-194-5p and miR-205-5p in b.End5 and 4T1 cells in the in vitro model of BCBM formation. ISH was performed using a 5′-3′ double digoxygenin (DIG)-labelled probe, containing locked nucleic acid (LNA) and 2′-O-methyl (2′OMe) RNA modified oligonucleotides (Qiagen, Dusseldorf, Germany). Fixed cells were permeabilised with 0.3% Triton X-100 for 15 min on ice and hybridised with correspondent probes (50 nM) at the hybridisation temperature of each miRNA for 60 min. The hybridisation signal was detected by adding alkaline phosphatase (AP)-labelled anti-DIG (1:1500, Roche, Basel, Switzerland) for 60 min at room temperature. Nitro-blue tetrazolium chloride (NBT)/5-bromo-4-chloro-3′-indolyphosphate p-toluidine salt (BCIP) (1:50, Roche, Basel, Switzerland) was used as a chromogenic substrate for AP. Nuclei counterstaining was performed with Hoechst 33342 dye (20 µM), for 10 min at room temperature. Negative control assays were performed without probes.

### 2.8. Image Acquisition

An automated HTS microscopy approach was used to evaluate the potential protective effect of each drug on the AJs protein β-catenin. Images from five different positions per well were acquired using a 40× 1.10NA water-immersion objective, using a Leica DMI6000B fully automated inverted fluorescence microscope equipped with a EL60000 metal halide light source (Leica Microsystems, Wetzlar, Germany) and a 2048 × 2048 pixel Orca-Flash4.0 CMOS camera (Hamamatsu, Hamamatsu, Japan).

ZO-1, p-MLC, and RhoA immunolabellings were examined using an Olympus BX60 microscope equipped with Olympus U-RFL-T Mercury lamp and Hamamatsu Orca R2 cooled monochromatic CCD camera, using a 40× oil objective. Ten fields of each cell culture condition were evaluated.

Coloured images of ISH were acquired using an Olympus BX51 bright field microscope with an integrated digital camera (Olympus, model DP50) with mercury fluorescence illuminator, and Nomarski/DIC Prism for Transmitted Light, using a 40× objective. Ten fields of each cell culture condition were evaluated.

Widefield images of the wound-healing assay were acquired after 0, 24, and 48 h, using a 10× objective with a phase contrast microscope (Nikon ECLIPSE TS100, Jenoptik) equipped with Nikon ELWD camera. Three technical replicates were performed and one image per well was acquired.

### 2.9. Image Analysis

Immunofluorescence images obtained by automated HTS or brightfield or fluorescence microscopy were examined using Icy (Institute Pasteur and France BioImaging, Paris, France) and ImageJ (National Institutes of Health, Bethesda, MD, USA) software. 

Membrane, nuclei, and total cell fluorescence intensity were quantified using three cells per image in which the area, ellipse, and polygon tools were used, respectively, in Icy software. Total membrane intensity/total cell intensity was calculated through the ratio of total circled membrane intensity and total circled cell intensity. The plot profiles for β-catenin localisation were obtained by the plot profile tool in ImageJ software.

For ZO-1, both membrane and cell mean fluorescence intensity, as well as membrane gaps, were quantified in five cells per image, using Icy software. Additionally, p-MLC and RhoA mean intensity per 4T1 cluster were quantified using ImageJ software, and 4T1 cell morphological parameters (elongation and roundness) were evaluated in five cells per image, using Icy software.

Tumour adherent cell analysis was made by counting the number of 4T1 cells per image, using the CellCounter tool in ImageJ, and the results were normalised to the respective control. 

In the wound-healing assay, wound closure was quantified using the line tool in ImageJ software to measure the distance from the wound edges. Three lines per image were drawn, corresponding to three different parts of the image (two field edges and the midline) and the results are presented as wound closure percentage of control (0 h), using the following equation:

$$\%\;\mathrm{closure}=100-\left(\frac{\mathrm{distance}\;\mathrm{at}\;\mathrm{chosen}\;\mathrm{timepoint}\;\times 100\;}{\mathrm{distance}\;\mathrm{at}\;0\;h}\right).$$

### 2.10. Statistical Analysis

Results were analysed using GraphPad Prism^®^ 8.0.2 (GraphPad Software, San Diego, CA, USA) and are expressed as mean ± SEM. The results represent the average of three independent experiments (n = 3). For parametric data, two-tailed Student’s *t*-test (miRNAs data analysis) and one-way ANOVA with Dunnet’s (viability and scratch assays) or Sidak’s (HTS, 4T1’s elongation and RhoA’s intensity) multiple comparisons tests were performed for comparisons between conditions. For non-parametric data with an abnormal distribution (α = 0.05) a Mann–Whitney test (ZO-1 intensity and number of gaps analysis) or a Kruskal–Wallis test (4T1’s adhesion, 4T1 roundness and p-MLC intensity analysis) were performed for comparisons between conditions. Statistically significant differences were considered when *p* < 0.05.

## 3. Results

### 3.1. Six Drugs Presented No Toxicity to BMECs and Did Not Increase BCCs Viability

**Figure 1:**
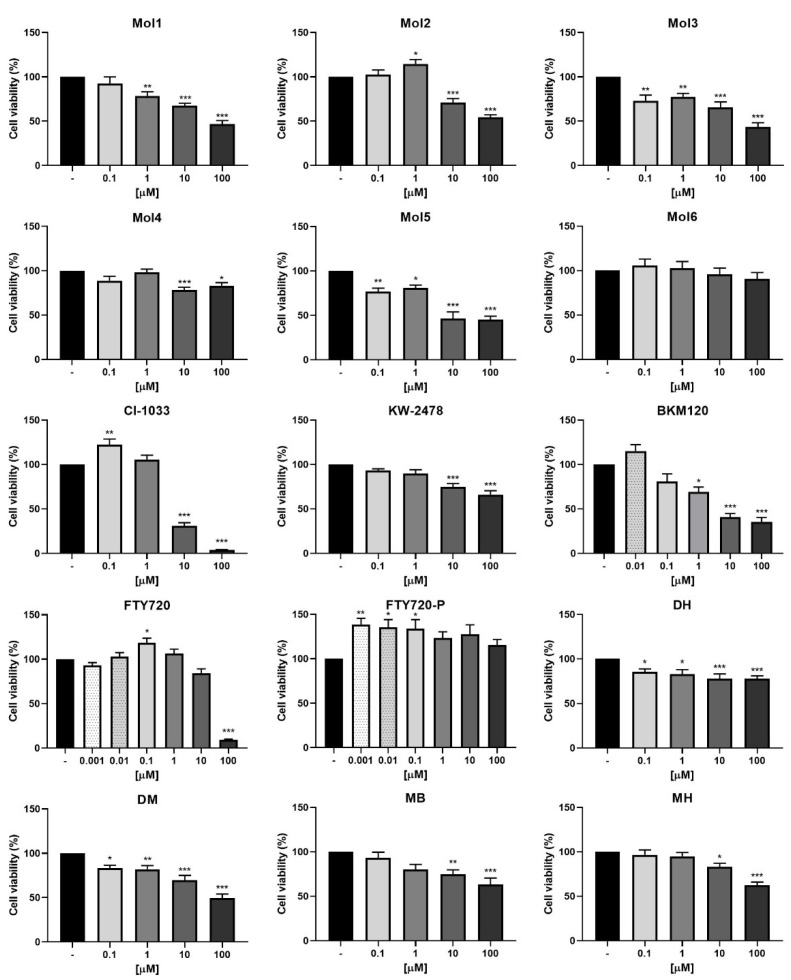
b.End5 cells viability upon exposure to drugs. B.End5 cells were treated with the indicated concentrations of each drug, or DMEM (untreated), for 24 h. Cell viability was assessed by the MTT assay, and the values are presented as percentage relative to untreated cells. Mol1, Mol2, Mol4, Mol6, CI-1033, KW-2478, BKM120, FTY720, FTY720-P, MB, and MH caused no toxicity at the lowest concentrations tested. All values are mean ± SEM of three independent experiments performed in triplicate. Statistical significances are shown as * *p* < 0.5, ** *p* < 0.01, and *** *p* < 0.001 vs. untreated.

As the major objective of this work is to act on the BBB, it is essential to ensure the safety of the drugs used to the brain microvascular endothelium. So, an initial viability assay by the MTT test was performed in b.End5 cells (Figure 1) to determine the safe concentrations of each of the 15 drugs. The results demonstrated that Mol3, Mol5, DH, and DM presented toxic effects at all the concentrations tested. Mol1 and BKM120 exhibited toxicity at 1 µM, while most of the drugs presented toxicity at concentrations ≥10 µM (Mol2, Mol4, CI-1033, KW-2478, MB, and MH), and FTY720 only presented toxicity at 100 µM. No toxic effect was observed at any concentration tested for Mol6 and for FTY720-P, which even increased cell viability at the lowest concentrations (0.001, 0.01, and 0.1 µM).

**Figure 2:**
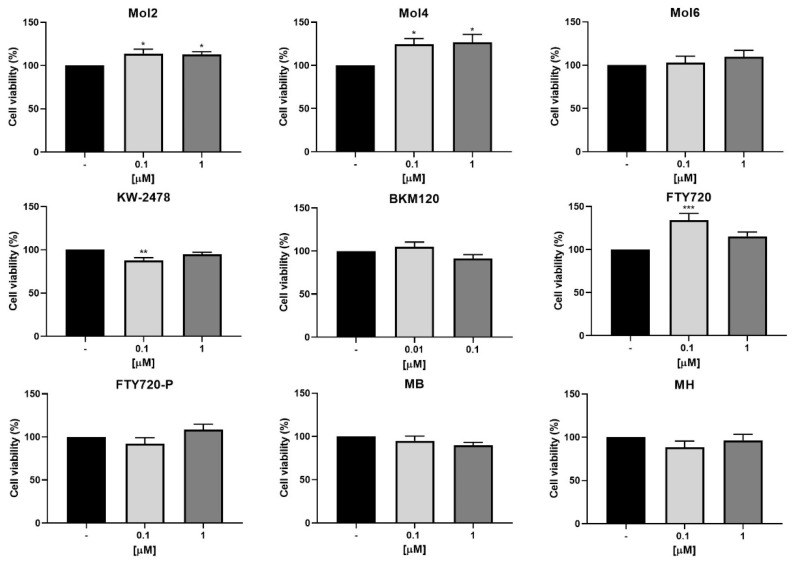
The 4T1 cells viability upon exposure to drugs. The 4T1 cells were treated with the indicated concentrations of each drug, or DMEM (untreated), for 24 h. Cell viability was assessed by MTT assay, and the values are presented as percentage relative to untreated cells. All values are mean ± SEM of three independent experiments performed in triplicate. Statistical significances are shown as * *p* < 0.05, ** *p* < 0.01, and *** *p* < 0.001 vs. untreated.

Based on the previous results, Mol2, Mol4, Mol6, KW-2478, BKM120, FTY720, FTY720-P, MB, and MH were selected for a further screen with 4T1 cells to ensure that they do not promote an increase in tumour cells’ viability (Figure 2). These drugs were tested at 0.1 and 1 µM except for BKM120 that was tested at 0.01 and 0.1 µM. The results demonstrated that Mol2 and Mol4 increase the tumour cells’ viability, with FTY720 only increasing BCCs viability at the lowest concentration, while Mol6, BKM120, FTY720-P, MB, and MH have no effect and KW-2478 decreases viability at 0.1 µM. Thus, Mol6, BKM120, FTY720-P, MB, MH, and KW-2478 were selected for the next phase to assess their capability to boost BBB properties, whereas those increasing 4T1 cell viability were excluded. From both concentrations of the selected drugs, the highest was the one chosen to ensure a visible effect in the following studies.

### 3.2. Effect of Several Drugs on Endothelial β-Catenin Expression

The six most promising drugs (Mol6, KW-2478, BKM120, FTY720-P, MB, and MH) were tested at the selected concentration for their ability to prevent the BBB endothelium disruption induced by exposure to tumour cells, as observed in previous work [40]. Considering that the AJs protein β-catenin is essential for BBB function and homeostasis [42] and a valuable indicator of barrier integrity [9], being expressed in BCCs with an epithelial phenotype [43], its expression and localisation were evaluated by immunofluorescence analysis in b.End5 and 4T1 cells, both in single and mixed cultures. 

#### 3.2.1. KW-2478 and Mol6 Increased β-Catenin Membrane Localisation in b.End5 Cells Exposed to BCCs

**Figure 3:**
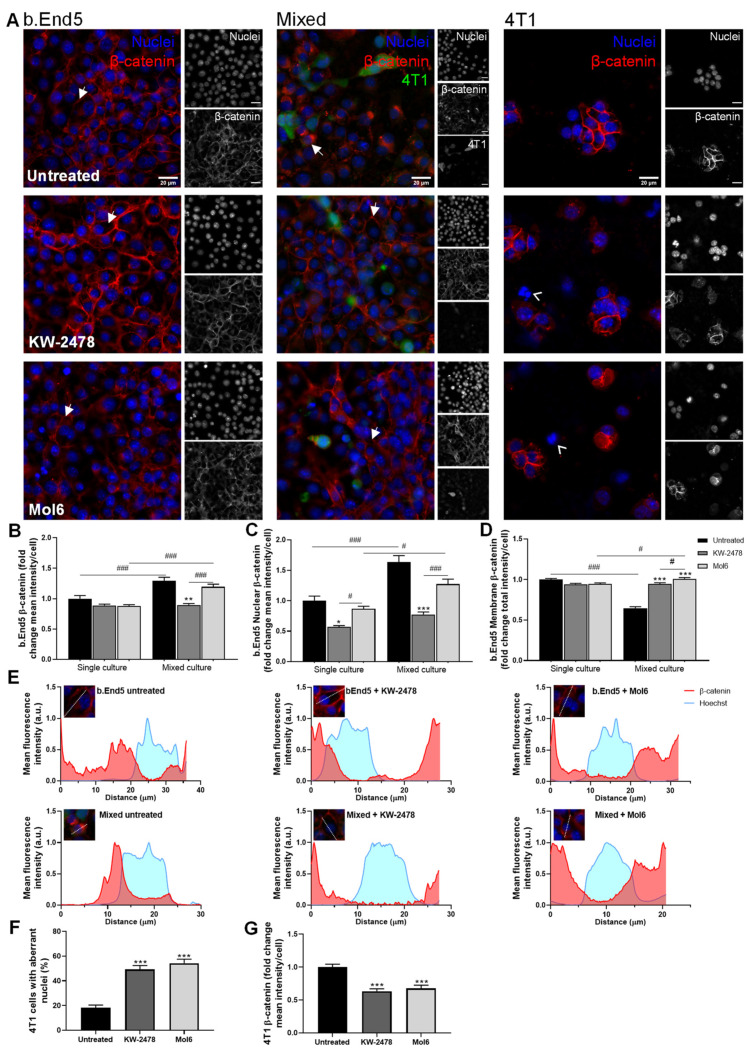
KW-2478 and Mol6 enhance barrier properties through increased membrane β-catenin expression in b.End5 cells exposed to 4T1 cells. Confluent monolayers of b.End5 cells under physiological laminar non-pulsatile shear stress were exposed to 4T1 cells (previously labelled with CellTracker™ CMFDA Green Dye,) and treated with KW-2478 or Mol6 (1 µM), or DMEM (untreated), for 24 h and the expression of the AJs protein β-catenin (red), in single and mixed cultures, was evaluated by immunofluorescence analysis. Hoechst 33342 was used as counterstaining for nuclei (blue). Scale bar: 20 µm. (**A**) Treatment with KW-2478 and Mol6 induced an increase in β-catenin expression in endothelial cells exposed to 4T1 cells, particularly at cell membrane (white arrows), and an increase in 4T1 cells with aberrant nuclei (white arrow heads). Semi-quantitative analysis of endothelial β-catenin (**B**) mean intensity per cell, (**C**) nuclear intensity, and (**D**) membrane intensity confirmed the observations. (**E**)The localisation of β-catenin was studied through plot profiles of pixel intensity throughout an endothelial cell (edge to edge). Semi-quantitative analysis of tumour cell cultures confirmed (**F**) an increase in the number of aberrant nuclei upon treatment with each drug, as well as (**G**) a decrease in β-catenin mean intensity per cell. Data are represented as mean ± SEM of three independent experiments, where 3 cells/field, 5 fields/condition were evaluated. Statistical significances are denoted as * *p* < 0.05, ** *p* < 0.01, and *** *p* < 0.001 vs. untreated conditions within the same culture type and ^#^ *p* < 0.05 and ^###^ *p* < 0.001 between indicated groups.

Alterations regarding β-catenin location and expression in the presence of the HSP90 and PI3K inhibitors, KW-2478 and Mol6, respectively, were monitored (Figure 3). b.End5 single cultures treated with KW-2478 or Mol6 showed no localisation changes in the expression of β-catenin, which maintained its expression at the membrane level, similarly to untreated cells. In mixed cultures, b.End5 monolayers presented a clear disruption, with loss of the AJs protein in the membrane and more expression in the cytoplasm and perinuclear region, together with 4T1 cells adherent in the existing holes between endothelial cells. Treatment with each of the inhibitors increased β-catenin expression at the membrane level, seemingly reducing b.End5 perinuclear β-catenin. Regarding 4T1 single cultures, both drugs induced alterations in nuclei morphology compared to untreated cells, compatible with cell impairment [44,45], and caused a reduction in the area of clustered cells (Figure 3A).

Semi-quantitative analysis revealed no differences in β-catenin mean intensity per cell value of single b.End5 cultures treated with each of the inhibitors compared to untreated b.End5 cells. However, an increase in β-catenin mean intensity per cell was observed in untreated (*p* < 0.001) and Mol6-treated mixed cultures (*p* < 0.001), which was not observed with KW-2478. Indeed, KW-2478 treatment kept these values similar to b.End5 untreated single cultures (Figure 3B). β-catenin nuclear mean intensity presented its lowest value in single cultures of b.End5 treated with KW-2478 (*p* < 0.05), and remained similar to control for Mol6 treatment. However, this intensity increased in untreated mixed cultures (*p* < 0.001) and in mixed cultures treated with Mol6 (*p* < 0.05), which was more evident in non-treated cultures. Treatment with KW-2478 resulted in the lowest values, which were similar to the ones observed in single cultures with the same treatment (Figure 3C). Of note, this increase in nuclear β-catenin intensity accompanied the previously described increase in β-catenin mean intensity per cell. Regarding the membrane β-catenin, both drugs induced a slight decrease in b.End5 single cultures compared to control. However, during exposure to 4T1 cells, both treatments kept membrane β-catenin similar to control and significantly higher than untreated mixed cultures (*p* < 0.001, Figure 3D). Those observations were validated by the plot profiles, which showed that from b.End5 single cultures to mixed cultures, β-catenin is present, particularly at the membrane level (Figure 3E). After addition of each drug, b.End5 cells recovered their membrane intensity compared to untreated mixed cultures, with a shift to membrane β-catenin localisation, especially noticeable with KW-2478 (Figure 3E).

Importantly, analysis of 4T1 single cultures revealed an increase in the percentage of cells with aberrant nuclei (i.e., fragmented, swollen, or blebbing nuclei) after treatment with both drugs (*p* < 0.001, Figure 3F), accompanied by the decrease in β-catenin fluorescence intensity (*p* < 0.001, Figure 3G), confirming the negative effect of these HSP90 and PI3K inhibitors towards TNBC cells.

#### 3.2.2. BKM120 Improves Barrier Properties via Increased β-Catenin Expression of b.End5 Cells in Single and Mixed Cultures

**Figure 4:**
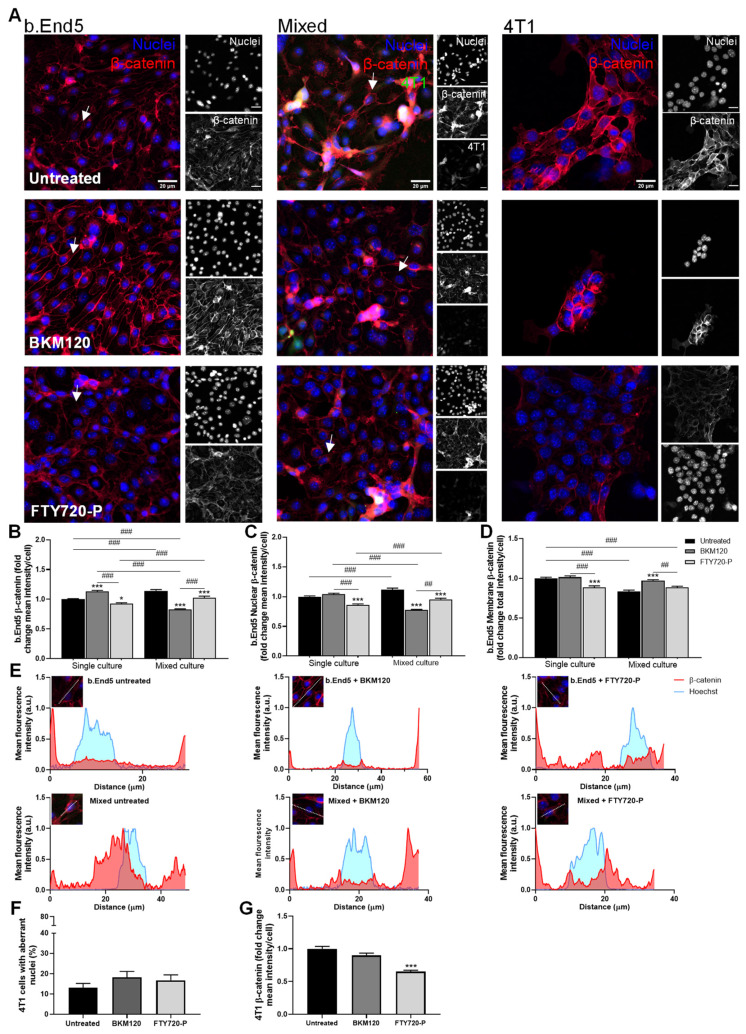
BKM120 induces endothelial barrier properties through the increase in membrane β-catenin expression. Confluent monolayers of b.End5 cells under physiological laminar non-pulsatile shear stress were exposed to 4T1 cells (previously labelled with CellTracker™ CMFDA Green Dye) and treated with BKM120 or FTY720-P (0.1 µM), or DMEM (untreated), for 24 h and the expression of the AJs protein, β-catenin (red), in single and mixed cultures was evaluated by immunofluorescence analysis. Hoechst 33342 was used as counterstaining for nuclei (blue). Scale bar: 20 µm. (**A**) Treatment with BKM120 or FTY720-P induced an increase in β-catenin expression in endothelial cells exposed to 4T1 cells, particularly at cell membrane (white arrows). Semi-quantitative analysis of endothelial β-catenin (**B**) mean intensity per cell, (**C**) nuclear intensity, and especially (**D**) membrane intensity validated these observations. (**E**) The localisation of β-catenin was studied through plot profiles of pixel intensity throughout an endothelial cell (edge to edge). Semi-quantitative analysis of tumour cell cultures revealed (**F**) no aberrant nuclei upon treatment, but (**G**) a decrease in β-catenin mean intensity per cell after FTY720-P treatment. Data are represented as mean ± SEM of three independent experiments, where 3 cells/field, 10 fields/condition were evaluated. Statistical significances are denoted as * *p* < 0.05 and *** *p* < 0.001 vs. untreated conditions within the same culture type and ^##^ *p* < 0.01 and ^###^ *p* < 0.001 between indicated groups.

Besides KW-2478 and Mol6, alterations regarding β-catenin location and expression in the presence of BKM120 or FTY720-P were evaluated (Figure 4). We observed that b.End5 single cultures treated with BKM120 showed a more organised monolayer and more notable membrane β-catenin expression than untreated b.End5 cells. FTY720-P caused an apparent decrease in β-catenin intensity and monolayer organisation, although maintaining most of the β-catenin expressed in the membrane. Treatment of mixed cultures with BKM120 allowed b.End5 cells to maintain β-catenin expressed mainly at the membrane, revealing fewer cells with cytoplasmatic or perinuclear β-catenin. On the other hand, b.End5 cells in mixed culture treated with FTY720-P had β-catenin expressed at the membrane level, though several cells also presented cytoplasmatic and perinuclear expression. Regarding 4T1 single cultures, BKM120 appeared to reduce the number of cells per cluster, while FTY720-P, despite apparently decreasing β-catenin expression, seems to increase the number of tumour cells per cluster (Figure 4A). 

Through semi-quantitative analysis of b.End5 cells, it was possible to confirm that BKM120 significantly increased β-catenin mean intensity per cell in single cultures (*p* < 0.001), while FTY720-P slightly decreased these values (*p* < 0.05). In untreated mixed cultures, b.End5 presented increased mean intensity per cell (*p* < 0.001), but treatment with BKM120 and FTY720-P kept these values lower (Figure 4B). The increased values observed in untreated mixed cultures were, once again, coincident with a peak in nuclei mean intensity per cell. Both treatments decreased nuclei intensity in mixed cultures (*p* < 0.001), with the lowest value observed in the cells treated with BKM120. In single cultures, FTY720-P reached the lowest nuclei mean intensity values (*p* < 0.001, Figure 4C). Regarding the membrane β-catenin, b.End5 single cultures treated with BKM120 kept values similar to control, but cells treated with FTY720-P suffered a decrease in membrane intensity (*p* < 0.001). b.End5 cells in untreated mixed culture faced a decrease in membrane intensity (*p* < 0.001), which was recovered with BKM120 treatment (*p* < 0.001), reaching values similar to control. However, FTY720-P did not seem to increase membrane intensity (Figure 4D).

Plot profiles of pixel intensity analysis of β-catenin localisation showed that, similarly to b.End5 untreated cells, cells treated with BKM120 presented a peak in β-catenin intensity in the membrane. As far as FTY720-P-treated cells are concerned, although having β-catenin in the membrane, they presented staining in the cytoplasm and nuclei as well. Untreated b.End5 in mixed cultures had more cytoplasmic β-catenin, especially perinuclearly and in the nuclei, rather than in the membrane. The effect of BKM120 in mixed cultures was notorious, keeping most of this protein located in the membrane, whereas FTY720-P maintained the majority of the protein at the cytoplasm (Figure 4E). 

Regarding tumour cells, neither of the drugs significantly affected the percentage of 4T1 cells with aberrant nuclei (Figure 4F). However, FTY720-P decreased β-catenin mean intensity per cell compared to untreated cells (*p* < 0.001), whereas treatment with BKM120 revealed no changes (Figure 4G).

BKM120 was selected for further studies due to its β-catenin-enhancing effect in b.End5 cells observed in single and, most importantly, in mixed cultures.

#### 3.2.3. MH Improves Barrier Properties through Increased β-Catenin Expression in b.End5 in Mixed Cultures

**Figure 5:**
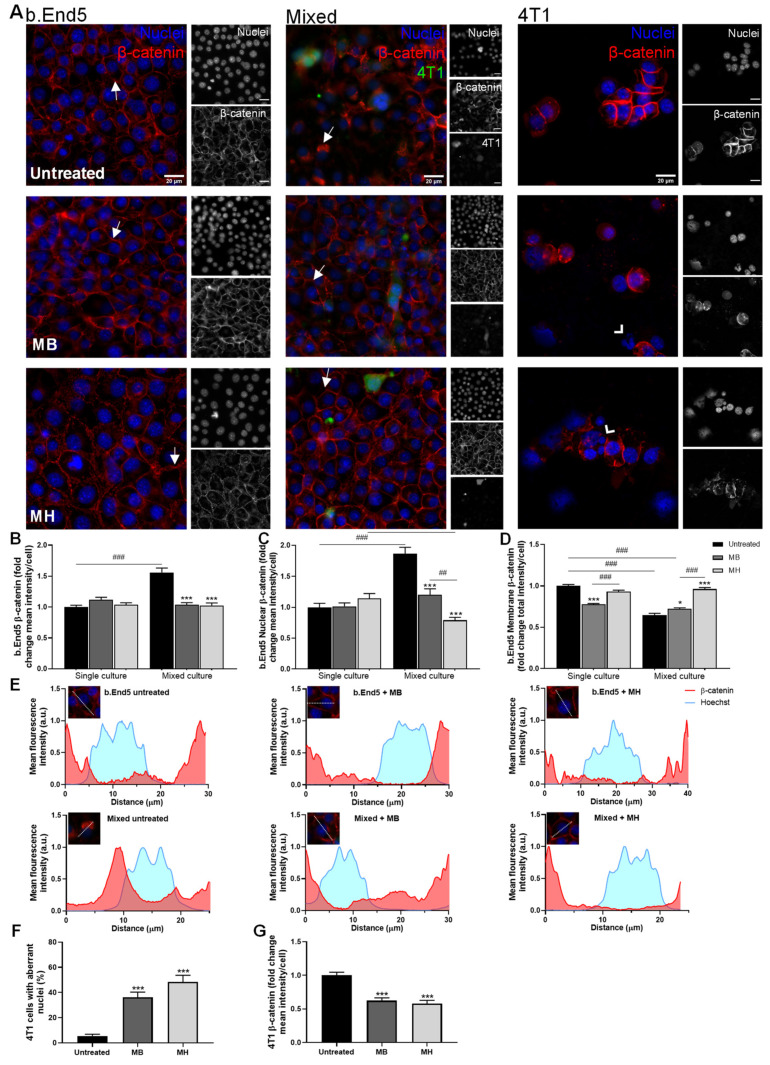
MH induces endothelial barrier properties through the increase in membrane β-catenin expression. Confluent monolayers of b.End5 cells under physiological laminar non-pulsatile shear stress were exposed to 4T1 cells (previously labelled with CellTracker™ CMFDA Green Dye) and treated with MB or MH (1 µM), or DMEM (untreated), for 24 h and the expression of the AJs protein β-catenin (red) in single and mixed cultures was evaluated by immunofluorescence analysis. Hoechst 33342 was used as counterstaining for nuclei (blue). Scale bar: 20 µm. (**A**) Treatment with MB and MH induced an increase in β-catenin expression in endothelial cells exposed to 4T1 cells, particularly at cell membrane (white arrows), and an increase in 4T1 cells with aberrant nuclei (white arrow heads). Semi-quantitative analysis of endothelial β-catenin (**B**) mean intensity per cell, (**C**) nuclear intensity, and especially (**D**) membrane intensity confirmed the observations. (**E**) The localisation of β-catenin was studied through plot profiles of pixel intensity throughout an endothelial cell (edge to edge). Semi-quantitative analysis of tumour cell cultures revealed (**F**) an increase in aberrant nuclei upon treatment with each drug, as well as (**G**) a decrease in β-catenin intensity per cell. Data are represented as mean ± SEM of three independent experiments, where 3 cells/field, 10 fields/condition were evaluated. Statistical significances are denoted as * *p* < 0.05 and *** *p* < 0.001 vs. untreated conditions within the same culture type and ^##^ *p* < 0.01 and ^###^ *p* < 0.001 between indicated groups.

Regarding tetracyclines (Figure 5A), we observed that the treatment of single cultures of b.End5 cells with MB did not affect endothelium organisation, while MH treatment induced a more organised phenotype of the endothelial monolayer. Like the other most promising drugs, tetracyclines MB and MH kept β-catenin visually expressed in the membrane in mixed culture. Moreover, both treatments seemed to have a negative effect in 4T1 cells alone, indicated by the nuclear features that suggest cell death.

Semi-quantitative analysis of β-catenin expression revealed no differences in β-catenin mean intensity per cell in single culture, though an increase in β-catenin mean intensity was observed in b.End5 cells in mixed cultures (*p* < 0.001), which was prevented by tetracycline treatment (*p* < 0.001, Figure 5B). As previously, the high β-catenin fluorescence intensity values observed in untreated mixed cultures are coincident with high nuclei intensity values (*p* < 0.001), though the nuclei mean intensity remained unchanged in single b.End5 cells exposed to both treatments (Figure 5C). Regarding membrane β-catenin, single b.End5 cultures suffered a decrease with MB treatment (*p* < 0.001), while for MH the values remained similar to untreated cells. However, the recovery of membrane β-catenin during exposure to 4T1 was remarkable with MH treatment (*p* < 0.001), but not so marked with MB treatment (*p* < 0.05; Figure 5D). The same was observed by localisation study, which confirmed that MB treatment caused single b.End5 cells to have β-catenin also located in the cytoplasm, in addition to the membrane; on the other hand, with MH treatment most of the β-catenin was concentrated in the membrane and in the regions of the cytoplasm closer to the membrane (Figure 5E). In mixed cultures, nuclei intensity was significantly lower when treated with any tetracycline, especially with MH treatment (*p* < 0.001, Figure 5C). This was supported by localisation analysis where a relocation of β-catenin occurs, which ceases to be in the perinuclear and nuclear region of the cell and passes essentially to the membrane (Figure 5E). Once more, the localisation study showed that MB treatment increased the localisation of this protein in the membrane, although also remaining in the cytoplasm. Notably, MH treatment maintained β-catenin localisation similar to untreated cells (Figure 5E).

Treatments with both MB and MH seem to negatively affect 4T1 cells, as suggested by the higher percentage of cells with aberrant nuclei (*p* < 0.001, Figure 5F) and lower β-catenin expression (*p* < 0.001, Figure 5G). 

Overall, KW-2478, BMK120, and MH were able to improve the expression of β-catenin in endothelial cells both in single and mixed culture, therefore being selected for the subsequent studies.

### 3.3. BKM120 and MH Reduce BCCs Adhesion to the Brain Endothelium

**Figure 6:**
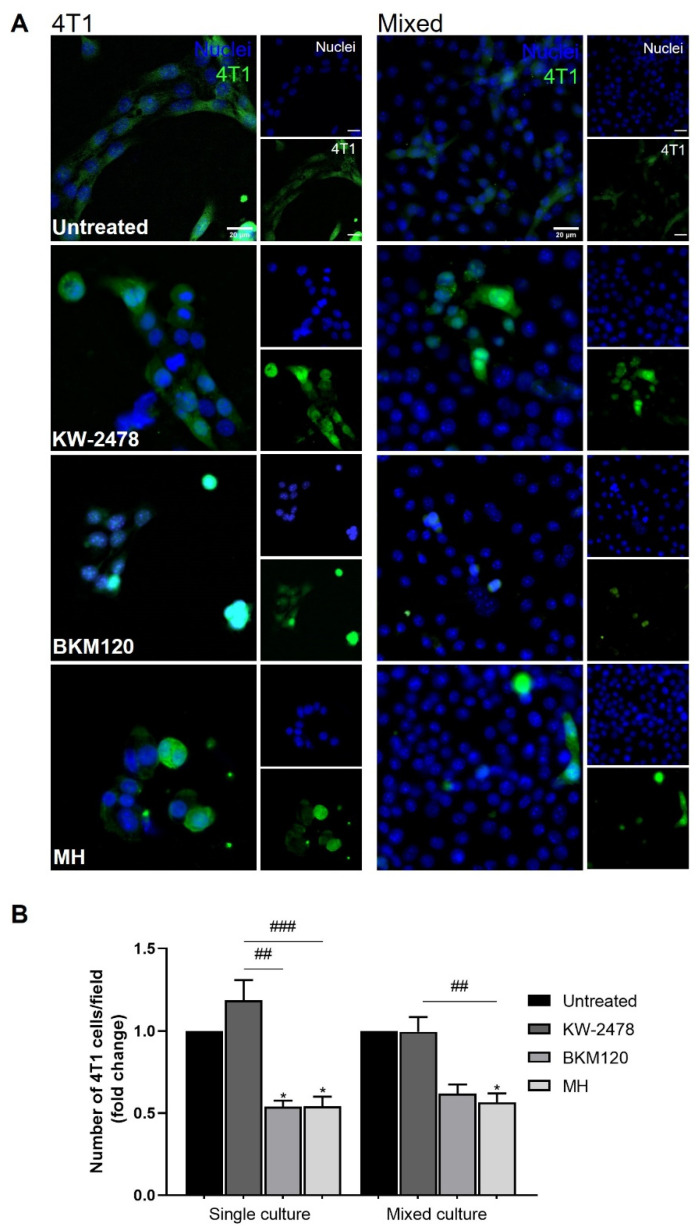
BKM120 and MH decrease the number of adherent 4T1 cells. Confluent monolayers of b.End5 under physiological laminar non-pulsatile shear stress were exposed to 4T1 cells (previously labelled with CellTracker™ DMFDA Green Dye) and treated with BKM120 (0.1 µM), KW-2478 (1 µM), MH (1 µM), or DMEM (untreated), for 24 h, and the adhesion of 4T1 in single and in mixed cultures was evaluated. Hoechst 33342 was used as counterstaining for nuclei (blue). Scale bar: 20 µm. (**A**) Immunofluorescence analysis revealed that BKM120 and MH promote the decrease in 4T1 adherence, particularly to the endothelium, confirmed by (**B**) quantitative analysis of 4T1 adherent cells, normalised to the respective control (untreated cells). Data are represented as mean ± SEM of three independent experiments, where 10 fields/condition were evaluated. Statistical significances are denoted as * *p* < 0.05 for treated vs. untreated conditions between the same culture type and ^##^ *p* < 0.01 and ^###^ *p* < 0.001 between the indicated conditions.

There is evidence supporting tumour cells’ necessity to adhere to brain endothelial cells to successfully overcome the BBB [46,47]. Thus, the number of adherent tumour cells can be an important parameter to obtain more information about the most promising drugs’ ability to prevent BCBM formation. In this sense, the number of adherent 4T1 cells per field was analysed for the three previously selected drugs, KW-2478, BKM120, and MH, after 24 h incubation (Figure 6). Through visual inspection of 4T1 cells in single culture, it was possible to observe that BKM120 and MH treatment decreased the size of cell clusters and the number of cells per field, while KW-2478 allowed the formation of bigger cell clusters and the adherence of a higher number of cells even though phenotypically affecting these cells (Figure 6A). Similarly, in mixed cultures, 4T1 were less adherent to the endothelium with BKM120 and MH treatment (Figure 6A).

By quantifying the number of 4T1 adherent cells per field, it was possible to observe that the drugs presented a similar effect on 4T1 behaviour in single and mixed cultures. Indeed, we observed that BKM120 and MH decreased the number of 4T1 adherent cells in single cultures (*p* < 0.05), with MH maintaining this profile in mixed cultures (*p* < 0.05), compared to their respective untreated control. KW-2478 revealed no significant changes in the number of adherent cells for any culture type (Figure 6B).

### 3.4. MH Strongly Inhibits BCCs Migration

Cell migration represents a hallmark of cancer invasion and metastases [43,48]. Intravasation, dissemination into the circulatory system, and extravasation of tumour cells are processes that culminate in metastatic growth and are highly dependent on the use of migration mechanisms [49]. Thus, migration assays, such as the wound-healing scratch assay, are good tools to assess the potential of the selected drugs in reducing the invasive properties of tumour cells.

**Figure 7:**
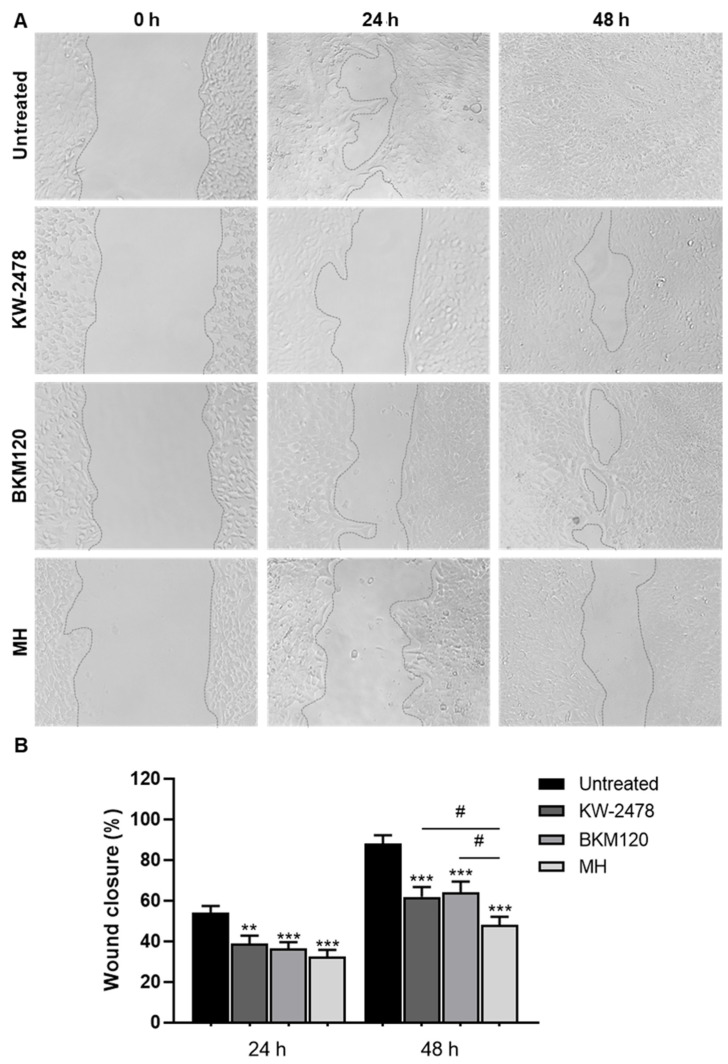
The 4T1 cell migration is inhibited by KW-2478 and BMK120 and especially by MH. The 4T1 cells were grown until confluence and then, after a scratch was made, incubated for 24 h or 48 h with BKM120 (0.1 µM), KW-2478 (1 µM), MH (1 µM), or DMEM (untreated). (**A**) Wound closure was monitored over time and images were acquired with a phase contrast microscope at 0, 24, and 48 h, demonstrating that MH was the drug that better inhibited 4T1 migration. (**B**) Quantitative analysis of wound closure presented as percentage of untreated cells. Data represented as means ± SEM of three independent experiments, performed in triplicate. Statistical significances are denoted as ** *p* < 0.01 and *** *p* < 0.001 vs. untreated conditions within the same culture type and ^#^ *p* < 0.05 between the indicated conditions.

The effects of the previously selected drugs (BKM120, KW-2478, and MH) were investigated at the same concentrations in BCCs migration through the wound-healing scratch assay (Figure 7). After 24 h of exposure to the drugs, 4T1 cells were significantly affected regarding their migration capacity. Visually analysing the images, it was possible to observe that untreated 4T1 cells began to close the wound at 24 h, already presenting some completely closed spots. Treatments with the drugs delayed wound closure for this timepoint, which did not present any closed site. After 48 h, untreated cells completely closed the wound. It was at this timepoint that the different effects of each drug were noticeable. In fact, BKM120 treatment presented some completely closed spots, but multiple others still to be closed; with KW-2478, the wound was also found to have already closed sites, especially at the ends of the scratch; however, MH treatment left the wound open, although with a smaller width compared to 0 h, appearing to be the drug with the greatest effect on the migration of these cells (Figure 7A). 

Quantitative analysis confirmed the drugs’ effect on 4T1 migration. Untreated 4T1 cells closed about 54% of the wound, compared to 39%, 36%, and 33% of KW-2478, BKM120, and MH, respectively, at 24 h. After 48 h, the effects between the different drugs were more visible. MH showed the lowest closure percentage (48%) among all drugs with a 40% difference compared to the untreated cells, and of about 16% and 14% compared to BKM120 and KW-2478, respectively. 

Taken altogether, MH appears as the most promising drug tested in our system and, therefore, was chosen for further assays. This drug not only has shown a beneficial effect on the endothelium exposed to tumour cells through the maintenance of optimal β-catenin expression, but also affected 4T1 negatively by increasing the number of cells with aberrant nuclei, decreasing β-catenin expression, and significantly modulating tumour cell adhesion and further inhibiting their migration.

### 3.5. MH Restores Tight Junctions’ Protein Expression

**Figure 8:**
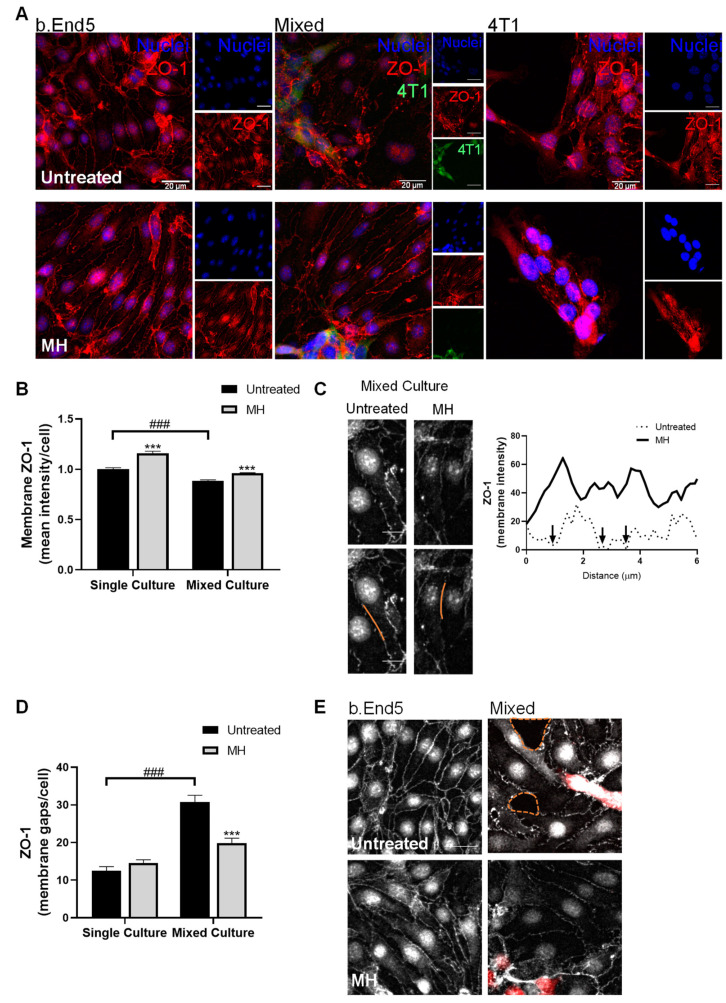
MH protects endothelial junctional properties. Confluent monolayers of b.End5 cells under physiological laminar non-pulsatile shear stress were exposed to 4T1 cells (previously labelled with CellTracker™ CMFDA Green Dye) and treated with MH (1 µM), or DMEM (untreated), for 24 h, and the expression of the TJs protein, ZO-1 (red), in single and mixed cultures, was evaluated by immunofluorescence analysis. (**A**) ZO-1 staining highlighted a clear expression in b.End5, particularly in cells treated with MH. Hoechst 33342 was used as counterstaining for nuclei (blue). Scale bar: 20 µm. (**B**) Semi-quantitative analysis showed an increase in membrane ZO-1 in b.End5 cells in single culture and that the contact with 4T1 cells induced a decrease, which was avoided by MH treatment. (**C**) Analysis of membrane ZO-1 expression (grey) in the orange region of the b.End5 cells in mixed culture revealed the presence of membrane gaps (black arrows in the plot) that were counteracted by MH treatment. Scale bar: 10 µm. (**D**) Semi-quantitative analysis of the number of membrane gaps in b.End5 cells corroborated the increase in mixed cultures and the decrease with MH treatment. (**E**) Inspection of the endothelial monolayer revealed holes (orange dotted lines) near 4T1 cells (red), which were absent upon MH treatment. Scale bar: 10 µm. Data are represented as mean ± SEM of three independent experiments, where 5 cells/field, 10 fields/condition were evaluated. Statistical significances are denoted as *** *p* < 0.001 vs. untreated conditions within the same culture type and as ^###^ *p* < 0.001 between the indicated conditions.

We next aimed to understand the ability of MH to further potentiate BBB properties upon TNBC cell exposure. To this end, an important TJs protein, ZO-1, described to be disrupted after contact with BCCs [40], was evaluated by immunofluorescence (Figure 8). 

Our results demonstrated that b.End5 cells express ZO-1 in both single and mixed cultures, in the presence or absence of MH, with an amelioration of ZO-1 expression and cell organisation in single and mixed cultures treated with MH being clearly noticeable (Figure 8A).

Semi-quantitative analysis revealed an increase in membrane ZO-1 fluorescence intensity in MH-treated b.End5 cells in single cultures (*p* < 0.001, Figure 8B). It also revealed a decrease in ZO-1 in b.End5 cells exposed to 4T1 cells (*p* < 0.001), which was restored in cells treated with MH (*p* < 0.001, Figure 8B). An in-depth analysis of ZO-1 expression in b.End5 exposed to 4T1 cells revealed that this TJs protein appeared discontinuous throughout the cell membrane, while in cells treated with MH the ZO-1 expression was more continuous (Figure 8C). Accordingly, semi-quantitative analysis showed an increase in the number of membrane gaps in b.End5 cells exposed to 4T1 cells (*p* < 0.001), which were partially prevented by MH treatment (*p* < 0.001, Figure 8D). Importantly, after contact with tumour cells, the endothelium appeared disrupted, as confirmed by the presence of holes in the monolayer, which were not observed in treated cells (Figure 8E). These observations support the protective role of MH in BBB integrity and proper junctions assembly.

### 3.6. MH Modulates b.End5 Elongation and BCCs Cytoskeleton

**Figure 9:**
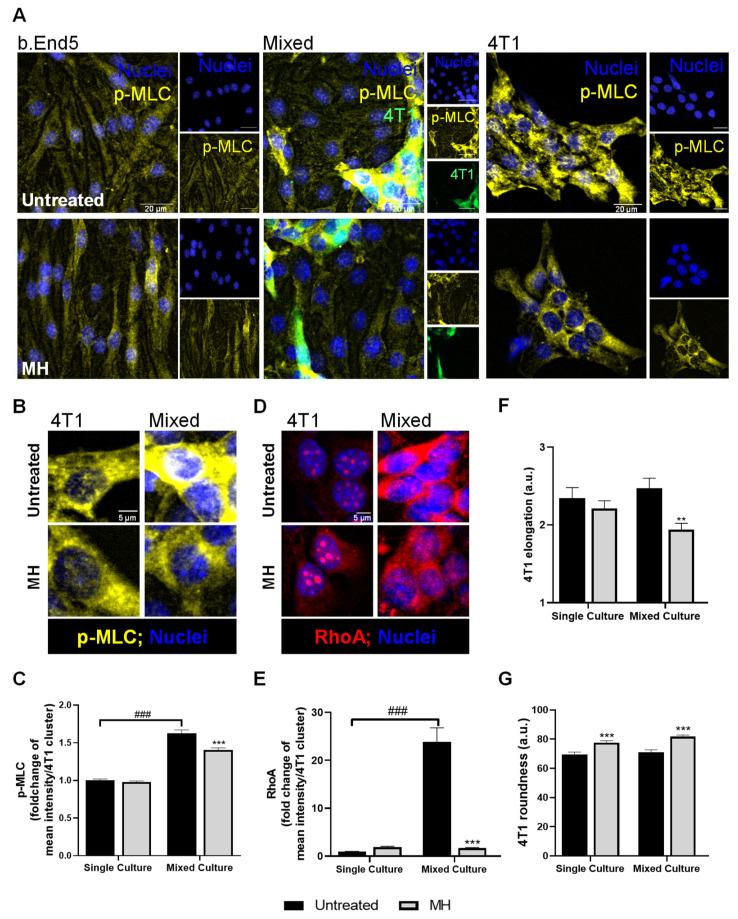
MH regulates endothelial and tumoural cytoskeleton-associated proteins. Confluent monolayers of b.End5 under physiological laminar non-pulsatile shear stress were exposed to 4T1 cells (previously labelled with CellTracker™ CMFDA Green Dye) and treated with MH (1 µM), or DMEM (untreated), for 24 h, and the cytoskeleton-associated alterations were evaluated in single and mixed cultures by immunofluorescence analysis of p-MLC (yellow) and RhoA (red). (**A**) p-MLC staining highlighted a clear expression in b.End5 and particularly in 4T1 cells within ‘metastasis-like’ clusters. Hoechst 33342 was used as counterstaining for nuclei (blue). Scale bar: 20 µm. (**B**,**C**) A significant decrease in tumour p-MLC in mixed cultures is observed upon treatment with MH. Scale bar: 5 µm. (**D**,**E**) RhoA staining highlighted a clear expression in 4T1 cell clusters, particularly in mixed cultures, which is counteracted by MH treatment, (**F**) reflecting a decrease in the elongated phenotype, and increasing (**G**) the roundness of tumour cells, coherent with a decrease in invasive properties. Scale bar: 5 µm. Data are represented as mean ± SEM of three independent experiments, where 5 cells/field, 10 fields/condition were evaluated. Statistical significances are denoted as ** *p* < 0.01 and *** *p* < 0.001 vs. untreated within the same culture type, and as ^###^ *p* < 0.001 between indicated conditions.

Next, we aimed to investigate the role of MH in endothelial cytoskeleton-associated proteins during TNBC cell transmigration across the BBB endothelium. To this end, p-MLC, described to be involved in cell contraction and endothelial hyperpermeability [50], as well as RhoA, described to be involved in tumourigenic processes [51], were evaluated by immunofluorescence (Figure 9).

We observed that b.End5 cells express p-MLC in single and in mixed cultures, both in the presence and absence of MH. The 4T1 cells abundantly express p-MLC in ‘metastasis-like’ clusters (Figure 9A). On the other hand, in mixed cultures, the 4T1 cells suffered an increase in p-MLC fluorescence intensity compared with 4T1 cells in single cultures, which was partially abolished upon treatment with MH (*p* < 0.001, Figure 9A–C). To further explore the possible impact of MH on the modulation of molecular mechanisms associated with tumour migration, the staining of RhoA was performed. As shown in Figure 9D and E, an increase in RhoA expression was observed in 4T1 cells upon contact with the endothelium (*p* < 0.001) that was thwarted by MH treatment (*p* < 0.001). Moreover, our data suggest a diminished elongation of tumour cells after treatment with MH (*p* < 0.01, Figure 9F), corroborated by the increased 4T1 cells’ round morphology (*p* < 0.001, Figure 9G).

### 3.7. MH Reverts BCBM Biomarkers Release and Expression

**Figure 10:**
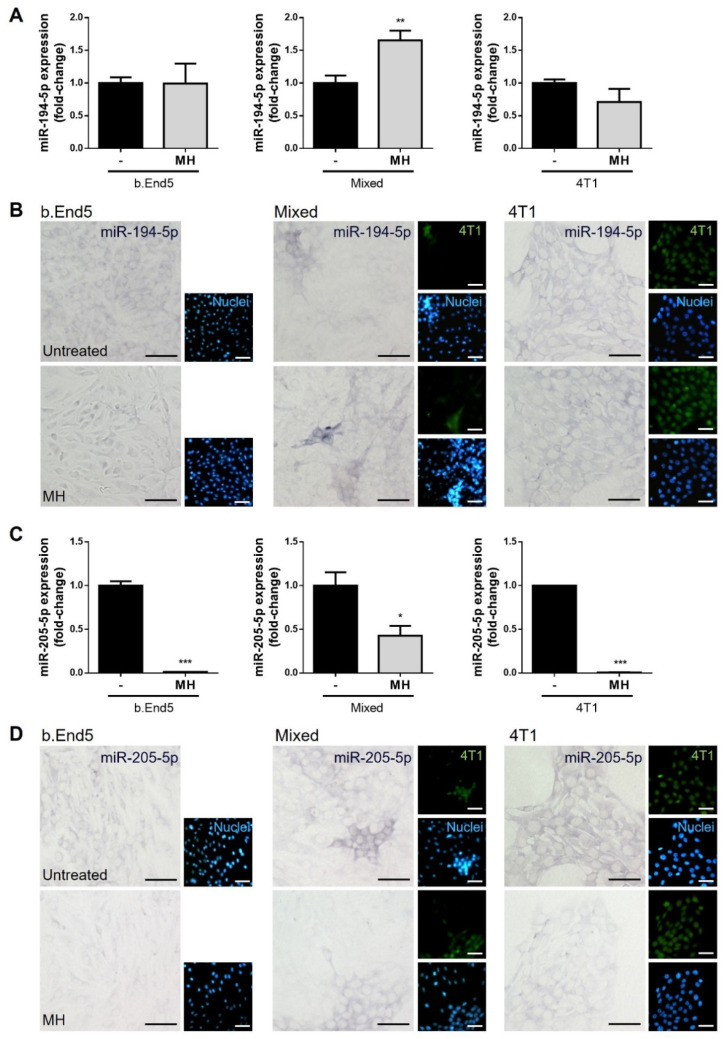
MH modulates miRNAs release and expression. Single cultures of 4T1, b.End5, and mixed cultures of b.End5 and 4T1 cells (previously labelled with CellTracker™ CMFDA Green Dye), under physiological shear stress, were treated with MH (1 µM), or DMEM (untreated), for 24 h, after which the media were collected and processed for RT-qPCR of the indicated miRNAs, while the cells were fixed and processed for ISH. (**A**) RT-qPCR analysis highlighted the increased miR-194-5p release in mixed cultures, (**B**) mirrored by its increased expression in b.End5 cells, especially in the vicinity of 4T1 clusters. (**C**) Downregulation of miR-205-5p release in single and mixed cultures with MH was observed by RT-qPCR, (**D**) as well as by ISH. RT-qPCR results are presented as fold change vs. untreated. Hoechst 33342 was used as counterstaining for nuclei (blue). Scale bar: 40 µm. Data are represented as mean ± SEM of three independent experiments. Statistical significances are denoted as * *p* < 0.05, ** *p* < 0.01, and *** *p* < 0.001 vs. untreated conditions.

In our previous studies, we found that BCBM formation is associated with a downregulation of miR-194-5p and an upregulation of miR-205-5p in plasma in early stages of BCBM formation, and that similar changes occur in cell cultures [37,38]. Aiming to understand if MH is able to modulate these miRNAs’ release into the cell medium and expression in each of the studied populations, RT-qPCR analysis of both miRNAs in cell medium and ISH in b.End5 and 4T1 single and mixed cultures, with and without MH, were performed (Figure 10).

MH treatment induced no alterations in single cultures but led to a significant increase in miR-194-5p release into the cell medium in mixed cultures (*p* < 0.01, Figure 10A), opposite to the miRNA downregulation observed both in vivo and in vitro [38]. Analysis of the miRNA in cells by ISH revealed that it is expressed by b.End5 and 4T1 cells, in single and mixed cultures, with and without MH. Comparably with the observations in the cell medium, an increase in bluish staining was evident in mixed cultures treated with MH, particularly in b.End5 cells in the vicinity of 4T1 clusters, pointing to an increased expression of this miRNA by b.End5 following drug treatment (Figure 10B).

Regarding miR-205-5p, treatment with MH promoted a significant decrease in its release by b.End5 (*p* < 0.001), by 4T1 (*p* < 0.001), and in mixed cultures (*p* < 0.05, Figure 10C). Similar alterations were observed among the different cell populations. Indeed, a decrease in miR-205-5p expression was observed in b.End5 and in 4T1 cells alone, as well as in mixed cultures, mainly in ‘metastasis-like’ clusters (Figure 10D), supporting the hypothesis that MH can modulate the expression of this miRNA, particularly in BCCs.

## 4. Discussion

**Figure 11:**
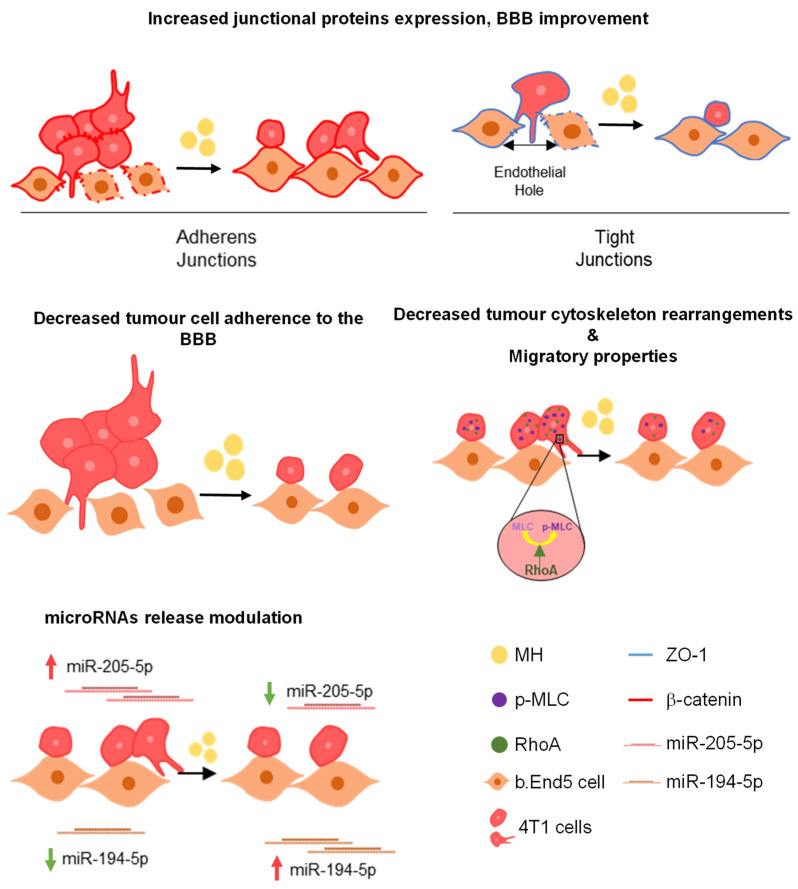
MH’s protective action on brain endothelium and inhibition of metastatic properties of tumour cells. MH is able to boost BBB properties and maintain the brain endothelial monolayer by improving AJs and TJs and preventing membrane gaps formation in b.End5 cells exposed to 4T1 cells. Moreover, MH is able to prevent 4T1 cells adhesion to brain endothelium and to decrease their migratory properties, apparently by decreasing p-MLC via downregulation of RhoA. MH is also able to prevent miR-205-5p and miR-194-5p changes, by decreasing the former and increasing the latter.

BC usually forms metastases in distant organs such as the brain, being associated with a poor prognosis [52]. Most of the studies are directed to primary tumours [53], whereas the modulation of BBB properties to inhibit BCCs extravasation into the brain and BCBM formation remains unstudied. The present work addresses the unmet need of discovering a drug able to boost BBB functions and inhibit the trafficking of tumour cells across it. The results ensuing from a screening of a library of molecules, including new drugs and approved ones, revealed MH’s ability to improve the BBB endothelium properties while disfavouring BCCs’ aggressiveness, as well as to counteract miR-194-5p and miR-205-5p deregulation (Figure 11). Therefore, this study paves the way for the repurposing of the clinically used antibiotic for prevention of brain metastasis formation in BC patients, and points to specific miRNAs as potential biomarkers of the treatment’s success.

Toxicity evaluation is an important step of preclinical safety assessment. Thus, a preliminary safety screening allowed the exclusion of drugs toxic for BMECs at low concentrations. It was also important to ensure that the drugs did not increase tumour cells viability. So, among the initial fifteen drugs, only six (Mol6, KW-2478, BKM120, FTY720-P, MB, and MH) met the criteria for further studies. Even so, this study raises the interest in further studying the anti-cancer effects of drugs such as Mol3, Mol5, CI-1033, DH, and DM, which showed toxicity against BMECs. Indeed, CI-1033 was described to have anti-proliferative properties against human epidermal growth factor 2 (HER2)-positive BCCs [54], while DH was described to ameliorate the number of BC stem cells positive for the proliferative marker, aldehyde dehydrogenase, in both HER2+ and TNBC cells [55]. Therefore, the molecules not meeting the inclusion criteria as drug candidates to prevent BCCs extravasation across the BBB endothelium may be candidates for treatment of BC, and particularly for BCBM, namely by using their encapsulation in adequate drug delivery systems specifically designed to act against BC and avoid collateral toxicity.

We took advantage of the combined use of automated and cell-based fluorescence microscopy to obtain non-biased images and study phenotypical changes in the AJs protein β-catenin. Moreover, we used an improved BBB in vitro model [40,56], encompassing physiological SS conditions, to more closely mimic the in vivo conditions [56]. Our results demonstrated that the presence of 4T1 cells significantly altered the expression of AJs, which, together with TJs, are known to have a crucial role in BBB integrity and homeostasis [9,57]. In fact, β-catenin expression in b.End5 cells exposed to 4T1 cells was more cytoplasmatic, nuclear, and perinuclear, rather than in the cell membrane, suggesting the loss of barrier function. BKM120, KW-2478, and MH were able to keep β-catenin expressed at the membrane level in mixed cultures, similarly to b.End5 cells in single culture. BKM120 and KW-2478’s roles in brain endothelial cells remain unstudied. BKM120 has only been described as possible therapy against primary and secondary tumours, namely BCBM [19,58,59]. Our studies showing that BKM120 enhances β-catenin expression in b.End5 cells, both in single and mixed cultures, open the possibility of using this PI3K inhibitor as a protective agent for the BBB. Besides being triggered in metastatic tumour cells, the PI3K pathway was also shown to be activated in brain endothelial cells that come into contact with cell-conditioned melanoma media [60]. In the present work, BKM120 enhanced β-catenin membrane expression in endothelial cells at a concentration of 0.1 µM, which is 10 to 20 times lower than the ones effective in other in vitro studies [58,59], raising its likelihood of being used in the future at lower clinical concentrations. As far as KW-2478 is concerned, it has only been studied against multiple myeloma, where it revealed antitumour properties in both in vitro and in vivo studies [23,24]. Our results go further by showing the potential of this drug to maintain β-catenin expression during exposure to tumour cells, as well as its negative effect in BCCs indicated by the increased number of cells with aberrant nuclei. The concentration used for this drug (1 µM) was lower than the effective ones in previous studies, which ranged from 3 to 5 µM [23,24]. Interestingly, MH’s results are in line with the literature showing that minocycline acts as an inhibitor of an upstream suppressor of Wnt/β-catenin signalling—Dickkopf-1 (DKK1)—promoting Wnt/β-catenin pathway activation, improving β-catenin levels in perihematomal tissues in an in vivo model of intracerebral haemorrhage [30]. Additionally, minocycline was shown to enhance the levels of TJs proteins, specifically ZO-1, occludin, and claudin-5, and to decrease the BBB permeability in an in vivo cerebral ischemia rat model, a condition that also compromises BBB function [31]. Even so, in a brain injury model, MH was shown to improve nerve function, and to ameliorate BBB damage associated with endoplasmic reticulum stress by boosting junctional proteins expression, particularly β-catenin [61].

To successfully extravasate across the BBB, tumour cells need to adhere to the endothelium, before performing transendothelial migration [43]. Indeed, real-time images of a mouse brain metastasis model revealed early extravasation and proximity to the microvasculature as key features for brain colonisation [46]. The selected drugs were further investigated through study of the number of adherent tumour cells to the endothelium. While KW-2478 did not affect the number of 4T1 adherent cells, BKM120 and MH significantly decreased tumour cells’ adhesion, both in mixed and single cultures. So, our results indicate that these drugs may inhibit the trafficking of tumour cells across the BBB, since adhesion precedes transmigration. 

BKM120 has previously revealed to block cell proliferation and cell cycle progression at concentrations of 0.5 and 1 µM [62]. In the present work, a concentration of 0.1 µM decreased the number of cells per field and reduced the size of cell clusters. Moreover, Mol6 and KW-2478, predicted PI3K [39] and HSP90 inhibitors, respectively, revealed a significant increase in the percentage of cells with aberrant nuclei, at a concentration of 1 µM. Interestingly, the concentrations of KW-2478 effective in other studies (e.g., in reducing tumour cell viability and/or proliferation) were slightly higher (3 to 5 µM) [23,24], compared with the ones used in this study (1 µM). On the other hand, MH has also been linked to decreased cell viability in human prostate and ovarian cancer cell lines [35,36], but not in TNBC cell lines [63]. Accordingly, 1 µM MH did not decrease 4T1 cell viability but led to an increasing number of cells with aberrant nuclei. Moreover, MH affected the phenotype of malignant cells, indicated by the reduced β-catenin expression. So, MH appears to have multiple effects, further discussed below.

To form metastases in distant organs, tumour cells need to increase their invasive and motility properties [64]. Therefore, the analysis of cell migration using simple and cost-effective methods such as an in vitro wound-healing assay represents a powerful tool [65]. Previous studies using PI3K inhibitors revealed the inhibition of BCCs migration [66]. The same inhibitory effect was particularly observed with BKM120 in glioma cells at concentrations ranging from 0.5 to 2 µM [59], supporting our observations. Indeed, the BKM120 delayed 4T1 cells’ migration in both timepoints, but after 48 h there were already some closed wound sites. It should be noted that BKM120 was used at a low concentration of 0.1 µM and still presented visible effects. Similarly, the HSP-90 inhibitor KW-2478 also inhibited the wound closure, though at a 10 times higher concentration. Interestingly, the greatest inhibitory effect in 4T1 migration was achieved with MH treatment, an effect currently unstudied in BCCs, and to our knowledge, this is the first time that such potential is reported for TNBC. Overall, our screening led us to choose MH for further studies, associated with enhancement of BBB properties and BCCs extravasation prevention.

To further explore and validate MH’s action on barrier properties, we evaluated the TJs protein ZO-1, described to be increased in an acute stroke animal model after treatment with MH [31]. Moreover, increased BBB properties with involvement of TJs expression have been shown to occur in response to the activation of the Wnt/β-catenin pathway by MH treatment [67]. In accordance, our results demonstrate that MH improves ZO-1 expression at the cell membrane and counteracts the appearance of monolayer holes in BMECs exposed to tumour cells, reinforcing the proneness of its barrier properties improvement. These observations are in line with previous ones demonstrating that minocycline improves BBB properties by upregulation of AJs and TJs proteins in an intracerebral haemorrhage model [30]. Moreover, they are supported by the previous observation of restoration of claudin-5 and ZO-1 levels upon MH therapy in animals with neuroinflammatory white matter injury [68]. Importantly, MH’s ability to counteract the disruptive effect observed in the presently used mouse model was also observed in a recently implemented human model of BCCs extravasation across the BBB (unpublished observations), indicating that the MH effect is not cell type dependent.

Cytoskeleton remodelling is one of the contributors to cell contraction and migratory properties [69]. In that sense, we evaluated the effect of MH in a cytoskeleton-associated protein, p-MLC, shown by our group to be related to endothelial changes during b.End5 cells’ exposure to 4T1 cells [40]. MH was able to partially restore endothelial cell elongation, supporting its protective effect in BBB endothelium. Of interest was the observation that, upon contact with the endothelium, tumour cells increase the phosphorylation of MLC, suggesting their increased cytoskeleton remodelling and enhanced migratory properties [70,71]. Indeed, myosin–actin contraction was associated with migration and invasion properties of tumour cells [72], and in particular of BCCs [73,74]. RhoA is an upstream player in cytoskeleton rearrangements, promoting the phosphorylation of MLC and cell contraction [51,75]. Accordingly, we demonstrated an upregulation of RhoA and p-MLC in 4T1 cells upon contact with the BBB endothelium, which was prevented by MH treatment. These results reinforce MH’s ability to inhibit BCCs’ migratory properties through modulation of cytoskeleton rearrangements via the RhoA/MLC pathway. Curiously, it was not the first time that this pathway was described to be modulated by clinically used pharmaceuticals [76]. In fact, both doxorubicin and paclitaxel modulated the RhoA/MLC pathway, although in an antagonistic way. In fact, doxorubicin increased the levels of RhoA and, consequently, MLC phosphorylation, promoting an increase in motility and migratory properties of BCCs, while paclitaxel led to their inhibition via a decrease in both RhoA and p-MLC levels [76]. RhoA has also been reported to be associated with the adhesion process of BC and melanoma cells, through the regulation of integrins and the capability of tumour cells to adhere to basal membrane components [77,78]. Those actions are in line with previous results of our group showing the interplay of β4-integrin with the adhesion and metastatic process of TNBC cells [40]. MH was able to restore the levels of RhoA and p-MLC in tumour cells in contact with the endothelium, suggesting a downregulation of adhesion and migratory and/or invasive properties. These results are corroborated by those of the scratch assay and of the number of adherent 4T1 cells, suggesting that pathways associated with cytoskeleton remodelling and adhesion are a target of MH.

During the extravasation process of BCCs across the BBB, some molecules, such as miRNAs, can be released into the circulation and putatively be used as biomarkers of disease and/or therapeutic response [37,38,79,80,81]. Moreover, some studies on cellular models of vascular proliferative diseases or cerebral ischemia have reported tetracyclines, in particular minocycline, as modulators of miRNAs expression [82,83]. Recently, our group identified miR-194-5p and miR-205-5p as potential biomarkers of BCBM [37,38]. To validate those miRNAs as possible biomarkers for therapeutic monitoring, we evaluated their release and cell content under MH treatment. We found that MH counteracts the deregulation in the release and expression of these miRNAs in both endothelial and tumour cells cultures, by upregulation of miR-194-5p and downregulation of miR205-5p. As far as we know, and despite the reports of minocycline as a miRNA expression modulator in some diseases, this is the first work on MH’s miRNA modulatory role in BCBM. Overall, our data point to the potential of these miRNAs as biomarkers of treatment response.

Last, but not least, minocycline has been used in cancer patients undergoing treatment with epidermal growth factor inhibitors as a prophylactic agent for dermatological toxicity, without causing side effects [84,85]. It was also used for symptom reduction in head and neck cancer patients, where it showed a positive outcome in decreasing symptom severity [86]. The doses used in the trials, ranging from 100 to 200 mg/day, had a safe profile, minimal side effects, and a serum half-life between 11 and 17 h [84,85,86]. In one particular trial, minocycline was administered in a dose of 200 mg twice daily [87]. For a 100 mg daily dose taken orally, serum minocycline concentrations ranged from 0.5 to 1.5 µg/mL; as for a 200 mg daily dose, serum concentrations comprehend values between 1.0 and 2.5 µg/mL [88]. Of note, the concentration used in our in vitro study (1 µM) is equivalent to 0.5 µg/mL, which can be observed in serum of patients administered with a 100 mg oral dose. However, we cannot ignore the fact that some studies suggest minocycline may not always be safe. Higher doses of minocycline increased neurotoxicity in in vivo models of neurodegeneration and neonatal hypoxia–ischemia conditions [89,90,91] and may have worsened outcomes in a clinical trial in amyotrophic lateral sclerosis [92]. Given this combined knowledge, minocycline can be a promising candidate for a targeted drug delivery system. In the past, MH was already encapsulated by liposome-based technology and successfully delivered to brain endothelial cells, revealing no toxicity in doses up to 7.5 µg/mL [93]; the challenge remains to disclose whether it would prevent BCBM formation in vivo.

Overall, our results support the choice of MH as a BBB modulator, acting as a BBB protective agent boosting AJs and TJs expression during the studied pathological condition. Moreover, it acts as an inhibitor of tumour cell migration and adhesion to the endothelium, confirming its possible anti-metastatic effect. Besides its effects at the junctional level and cytoskeleton-associated proteins, MH is further able to modulate TNBC brain metastases-related miRNAs, namely the downregulated miR-194-5p and the upregulated miR-205-5p, counteracting their release and expression.

## 5. Conclusions

Through a high-throughput microscopy screening of several new molecules and some approved drugs, our study identified buparlisib, KW-2478, and MH as promising molecules to act upon the BBB, enhancing its properties through modulation of an AJs protein expression, as well as to inhibit tumour cells adhesion and migration. Moreover, MH was disclosed as a potential BBB modulator, able to enhance AJs and TJs proteins expression at the membrane level, and to decrease monolayer disruption while maintaining endothelial cell morphology, during exposure to BCCs. Additionally, MH negatively affected BCCs, increasing the number of aberrant nuclei, suggestive of cell death. MH further decreased the number of adherent cells to the endothelium, pointing to its effect on preventing the tumour cells’ transmigration across the BBB endothelium. MH also significantly decreased BCCs migratory properties, altered cytoskeleton rearrangement, and modulated miRNAs expression and release. Our data provide advances beyond the current state of the art in the field of BCBM and, thus, in neurooncology, paving the way for the development of a novel therapeutic approach directed to the prevention of brain metastases formation. We highlight the fact that MH is already used in clinics and that its repurposing would speed up the implementation of the new therapeutics, with great benefits in prolonging the brain metastases-free survival of BC patients and, thus, their quality of life. Finally, MH’s ability to counteract BCBM-associated miRNAs deregulation should be noted, which opens the possibility of inferring the treatment efficacy based on specific miRNAs analysis in liquid biopsies.

## Figures and Tables

**Table 1:** Summary of the experimental conditions for immunofluorescence analysis.

Target Protein	Primary Antibody	Secondary Antibody
β-catenin	β-catenin (1:100) Thermo Fisher Scientific, #71-2700, Rabbit	Alexa Fluor^®^ 555 (1:500) Thermo Fisher Scientific, #A21428, Goat Anti-Rabbit
p-MLC	p-MLC (1:400) Thermo Fisher Scientific, #MA5-15163, Mouse	Alexa Fluor^®^ 555 (1:500) Thermo Fisher Scientific, #A31570, Donkey Anti-Mouse
RhoA	RhoA (1:100) Thermo Fisher Scientific, #OSR00266W, Rabbit	Alexa Fluor^®^ 555 (1:500) Thermo Fisher Scientific, #A21428, Goat Anti-Rabbit
ZO-1	ZO-1 (1:200) Thermo Fisher Scientific, #40-2200, Rabbit	Alexa Fluor^®^ 555 (1:500) Thermo Fisher Scientific, #A21428, Goat Anti-Rabbit

p-MLC, phosphorylated myosin light chain; RhoA, ras homolog family member A; ZO-1, zonula occludens-1.

## Data Availability

All data generated or analysed during this study are included in this published article.

## Supplementary Materials

The following supporting information can be downloaded at: https://www.mdpi.com/article/10.3390/biomedicines10081988/s1, Table S1: Summary of the molecules used and their molecular and structural features.
