# Supplementary material for: A Drug Screening Reveals Minocycline Hydrochloride as a Therapeutic Option to Prevent Breast Cancer Cells Extravasation across the Blood–Brain Barrier
Source: Biomedicines. 2022 Aug 16;10(8):1988. doi: 10.3390/biomedicines10081988 (PMC9405959; doi:10.3390/biomedicines10081988)
Supplement: Supplementary file 1 [file biomedicines-10-01988-s001.zip › biomedicines-1852939-supplementary.pdf]

Supplementary Data:

Supplementary Table S1. Summary of the molecules used and their molecular and structural features

Drug type	Drug	Abbreviation used	Zinc ID / CAS number	Molecular weight (g/mol)	Chemical Structure
PI3K inhibitor	Molecule 1	Mol1	ZINC20615563	518.4	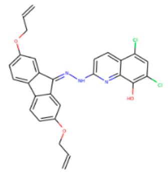
	Molecule 2	Mol2	ZINC36307506	475.6	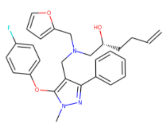
	Molecule 3	Mol3	ZINC9873787	477.6	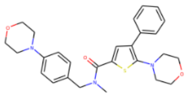
	Molecule 4	Mol4	ZINC977288	365.4	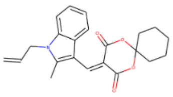
	Molecule 5	Mol5	ZINC1488208	369.2	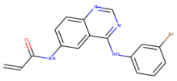
	Molecule 6	Mol6	ZINC218287337	423.9	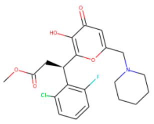
	Buparlisib	BKM120	ZINC43154039	410.39	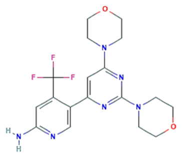
HSP 90 inhibitor	KW-2478	KW-2478	ZINC68202727	574.7	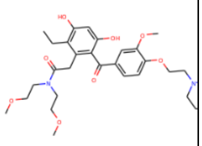

EGFR Tyrosine Kinase	Canertinib	CI-1033	ZINC27439698	485.9	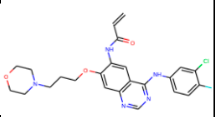
Sphingosine-1-phosphate receptor modulator	Fingolimod	FTY720	162359-56-0	343.93	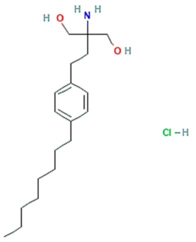
	Fingolimod-phosphate	FTY720-P	402615-91-2	387.45	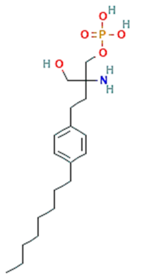
Tetracycline	Minocycline hydrochloride	MH	13614-98-7	493.9	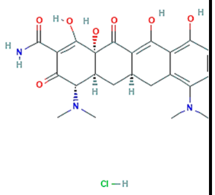
	Minocycline Base	MB	10118-90-8	457.48	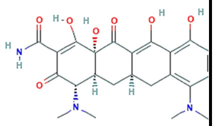
	Doxycycline Hyclate	DH	24390-14-5	1025.9	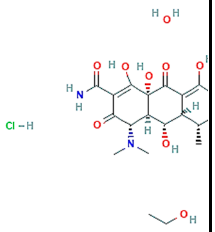
	Doxycycline Monohydrate	DM	17086-28-1	462.4	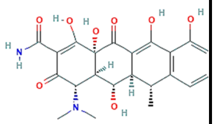
